# Genotoxicity, acute and subchronic oral toxicity assessments of postbiotics of *Lacticaseibacillus rhamnosus* IDCC 3201

**DOI:** 10.71150/jm.2605002

**Published:** 2026-06-12

**Authors:** Shin-Yae Choi, Dahae Hong, Jin Seok Moon, O-Hyun Ban, Hee-Won Bae, Tae-Yoon Kim, You-Hee Cho

**Affiliations:** 1 Department of Pharmacy, College of Pharmacy and Institute of Pharmaceutical Sciences, CHA University, Seongnam 13488, Republic of Korea; 2 Ildong Pharmaceuticals Co. Ltd., Seoul 06752, Republic of Korea; 3 Ildong Bioscience, Pyeongtaek 17957, Republic of Korea

**Keywords:** postbiotics, *Lacticaseibacillus rhamnosus* IDCC 3201, safety assessment, genotoxicity, oral toxicity

## Abstract

Postbiotics derived from lactic acid bacteria (LAB) have attracted growing interest as stable and potentially safer alternatives to probiotics for use in foods and health-related products. Comprehensive safety evaluation remains essential before their broader application. In this study, we assessed the safety profiles of RHT3201, a postbiotic preparation derived from *Lacticaseibacillus rhamnosus* IDCC 3201, through genomic, genotoxic, acute oral, and subchronic oral toxicity studies. Whole-genome analysis showed that IDCC 3201 lacks antimicrobial resistance genes and exhibits no hemolytic activity, supporting the genomic safety of the source strain. RHT3201 showed no genotoxic potential in either in vitro or in vivo assays, as evidenced by no structural or numerical chromosomal aberrations at concentrations up to 5,000 μg/ml in CHL/IU cells and no increase in micronucleated polychromatic erythrocytes, with no suppression of bone marrow erythropoiesis by oral administration of RHT3201 at doses up to 15,000 mg/kg/day using a mouse model. In rats, single oral doses of up to 15,000 mg/kg caused no mortality, treatment-related clinical signs, or gross pathological abnormalities, indicating an approximate lethal dose greater than 15,000 mg/kg. In a 90-day repeated-dose oral toxicity study, no adverse treatment-related effects were observed at doses up to 5,000 mg/kg/day. Mild liver and thyroid histopathological findings were considered adaptive and reversible. Accordingly, the no-observed-adverse-effect level was determined to be 5,000 mg/kg/day. Taken together, these findings support the safety of RHT3201 as a LAB-derived postbiotic ingredient.

## Introduction

Postbiotics, comprising non-viable microbial cells and their metabolites, have emerged as promising agents for promoting human health without the risks associated with live microorganisms (Aguilar-Toalá et al., 2018; Salminen et al., 2021). Lactic acid bacteria (LAB), including *Lactobacillus* and related genera, have long been recognized for their beneficial effects on host physiology. Numerous studies have demonstrated that LAB contribute to the maintenance of intestinal homeostasis by modulating the gut microbiota, enhancing epithelial barrier function, and regulating immune responses (Kim et al., 2017, 2026a; Lebeer et al., 2008; Lee at al., 2018; Ouwehand et al., 2002; Song et al., 2025). In particular, LAB are known to exert immunomodulatory effects by influencing the balance of pro- and anti-inflammatory cytokines, such as interleukin (IL)-10, IL-12, and tumor necrosis factor-α (TNF-α), thereby playing a role in the prevention of inflammatory and allergic diseases (Plaza-Díaz et al., 2017; Taverniti and Guglielmetti, 2011). In addition, LAB have been reported to exhibit antioxidant activity and to inhibit the growth of pathogenic microorganisms through the production of organic acids and antimicrobial compounds (Lebeer et al., 2008; Lin and Yen, 1999).

Traditionally, these health-promoting effects have been attributed to probiotics, which are defined as live microorganisms that confer health benefits to the host when administered in adequate amounts. However, the requirement for microbial viability poses several limitations (Hill et al., 2014; Sanders et al., 2010). The stability of probiotic products can be affected by storage conditions, processing, and gastrointestinal environments, leading to reduced viability and inconsistent efficacy. Moreover, the use of live microorganisms may raise safety concerns, particularly in immunocompromised individuals, where cases of bacteremia and opportunistic infections have been occasionally reported (Imperial and Ibana, 2016; Sanders et al., 2010). These limitations have prompted increasing interest in alternative approaches that can retain the beneficial effects of probiotics while minimizing associated risks, as exemplified by postbiotics defined as a preparation of inanimate probiotic microorganisms and/or their components that confers the beneficial effects of the probiotics (Aguilar-Toalá et al., 2018; Salminen et al., 2021). Nevertheless, comprehensive safety evaluation still remains a critical prerequisite for the development and commercialization of such postbiotic products. As postbiotics are derived from microbial sources, it is essential to ensure that they do not contain harmful genetic elements, such as antimicrobial resistance genes or virulence factors that could elicit pathogenic responses in the host, and that they do not induce genotoxic or systemic toxic effects upon administration. In particular, evaluating the genotoxic potential is crucial, as substances capable of damaging genetic material may lead to mutations and carcinogenesis (Kirkland et al., 2005; OECD, 2016a, 2016b). Regulatory frameworks, including those proposed by the European Food Safety Authority, emphasize the importance of assessing genomic safety, antimicrobial susceptibility, and toxicological profiles to establish the safety of microbial-derived products intended for human consumption (EFSA Scientific Committee, 2025). Accordingly, genotoxicity assays, as well as acute and repeated-dose toxicity studies, are considered essential components of safety assessment, providing insights into both the immediate and long-term effects of a substance following single or repeated exposure (OECD, 2008, 2018).

*Lacticaseibacillus rhamnosus* IDCC 3201 is a well-characterized strain originally isolated from a breast milk-fed infant and has been investigated for its potential health-promoting properties (Chae et al., 2022; Kwon et al., 2024). The tyndallized form of this strain, referred to as RHT3201, consists of heat-inactivated bacterial cells and culture-derived components, which may retain bioactive functionality while eliminating risks associated with microbial viability (Piqué et al., 2019). Previous studies have suggested that RHT3201 exhibits beneficial effects, including immunomodulatory and antioxidant activities, supporting its potential application as a functional ingredient (Kim et al., 2026b). In the present study, we evaluated the comprehensive safety profiles of RHT3201 through a multi-layered approach, including genomic analysis, genotoxicity assessment using in vitro and in vivo assays, and acute and subchronic oral toxicity studies in rodents. These investigations provide essential evidence supporting the safe use of RHT3201 as a postbiotic ingredient for potential applications in food and health-related products.

## Materials and Methods

### Bacterial strains and growth conditions

*L. rhamnosus* IDCC 3201 (ATCC BAA-2836) was cultured in whey-based medium at 37°C for 16 h. Bacterial cells and culture supernatant were separated by centrifugation (8,000 rpm, 10 min). The supernatant was concentrated using a vacuum evaporator and mixed with cornstarch powder (Daesang, Korea) at a 2:1 ratio. Bacterial pellets were heat-inactivated at 70°C for 2 h. Both fractions were lyophilized separately and subsequently combined to obtain the final product (RHT3201), containing 2.5 × 10^10^ CFU/g of inactivated cells and 120 mg/g of lactic acid.

### Whole genome sequencing and strain identification

Genomic DNA was extracted using the DNeasy Blood & Tissue Kit (Qiagen, Germany), and DNA quality and concentration were assessed using a NanoVue spectrophotometer (GE Healthcare, UK). Whole genome sequencing (WGS) was performed using a hybrid approach combining PacBio RS II (Pacific Biosciences, USA) and Illumina MiSeq (Illumina, USA) platforms. Libraries were prepared using the Nextera DNA Sample Preparation Kit and Index Kit (Illumina, USA), followed by purification with AMPure XP beads (Beckman Coulter, USA). Sequence reads were assembled using HGAP3 and corrected using Pilon. The obtained genome sequence (accession number: NZ_CP045531) was compared with reference strains of *L. rhamnosus*, including HN001 (accession number: NZ_CP174514) and GG (accession number: NZ_CP031290).

### Antibiotic susceptibility test

Antibiotic susceptibility was determined by the broth microdilution method according to CLSI guidelines (CLSI, 2026). The following antibiotics were tested: ampicillin (Sigma-Aldrich, USA), chloramphenicol (Sigma-Aldrich, USA), clindamycin (Sigma-Aldrich, USA), erythromycin (TCI, Japan), gentamicin (Sigma-Aldrich, USA), kanamycin (Sigma-Aldrich, USA), streptomycin (TCI, Japan), tetracycline (Sigma-Aldrich, USA), and vancomycin (Sigma-Aldrich, USA). MIC values were interpreted as described elsewhere (Kim et al., 2024).

### Virulence associated gene analysis

The search for virulence factors in WGS of IDCC 3201 was completed using the Virulence Factors Database hosted by Institute of Pathogen Biology (http://www.mgc.ac.cn/) (Liu et al., 2019). Additionally, the WGS of *L. rhamnosus* IDCC 3201 was compared with virulence genes of four pathogens (*E. coli*, *Enterococcus*, *Listeria*, and *Staphylococcus aureus*) using VirulenceFinder2.0 hosted by the Center for Genomic Epidemiology (http://www.genomicepidemiology.org/) (Joensen et al., 2014).

### Hemolysis

Hemolytic activity was evaluated using blood agar base (MB Cell, USA) supplemented with 1.5% Bacto agar (BD Difco, USA) and 7% sheep or horse blood (MB Cell, USA). Plates were incubated at 37°C for 48 h under anaerobic conditions. Hemolytic patterns were classified as α-, β-, or γ-hemolysis based on the presence or absence of hemolytic zones. *Streptococcus pneumoniae* ATCC 6305, *Staphylococcus aureus* ATCC 25923, and *Enterococcus faecalis* ATCC 29212 were used as controls.

### Dose formulation and analysis

Water for injection served as the vehicle. The test item was suspended in the vehicle to prepare homogeneous dosing formulations, sampled from the top, middle, and bottom to confirm homogeneity. Formulations at 10 and 500 mg/ml were stable and homogeneous for 4 h at room temperature and for 8 days refrigerated (2–8°C). Lactic acid, a marker compound of RHT3201, was analyzed by UPLC (ACQUITY UPLC, Waters, USA). Concentrations were verified before dosing. Analyses were performed once for the in vitro chromosomal aberration assay, micronucleus assay in mice, and acute toxicity study in rats, and twice (before the first and last treatments) for the subchronic toxicity study in rats.

### In vitro chromosomal aberration assay

The in vitro chromosomal aberration assay was conducted using Chinese hamster lung (CHL/IU) cells (ATCC, USA) in accordance with OECD Test Guideline 473 (OECD, 2016a). Cells were treated with RHT3201 at concentrations of 0, 1,250, 2,500, and 5,000 μg/ml, with or without metabolic activation (S9 mix, Aroclor 1254-induced rat liver). Mitomycin C and benzo[a]pyrene were used as positive controls. Chromosomal aberrations were evaluated from 300 metaphase cells per dose.

### Micronucleus assay in mice

The in vivo micronucleus assay was performed in male ICR mice (Samtako Inc., Korea) following OECD Test Guideline 474 (OECD, 2016b). RHT3201 was administered orally at doses of 0, 3,750, 7,500, and 15,000 mg/kg. Mitomycin C (Sigma-Aldrich, USA) was used as a positive control. Bone marrow cells were collected 24 h after the final administration and analyzed for micronucleated polychromatic erythrocytes (MNPCEs) and the PCE/NCE ratio.

### Acute oral toxicity study in rats

Acute oral toxicity was evaluated in male and female Sprague-Dawley (SD) rats (Samtako Inc., Korea) in accordance with OECD Test Guideline 420 (OECD, 2008). RHT3201 was administered orally at doses of 0, 1,500, 5,000, and 15,000 mg/kg. Animals were observed for 14 days for mortality, clinical signs, and body weight changes, followed by gross necropsy.

### Subchronic oral toxicity study in rats

Subchronic toxicity was assessed in male and female SD rats (Samtako Inc., Korea) following OECD Test Guideline 408 (OECD, 2018). RHT3201 was administered orally at doses of 0, 800, 2,000, and 5,000 mg/kg/day for 90 days, followed by a 28-day recovery period. Clinical observations, body weight, food consumption, clinical pathology, organ weights, and histopathological examinations were conducted.

## Results

### Genomic safety assessment of the source strain

To verify the intrinsic safety of *Lacticaseibacillus rhamnosus* IDCC 3201 at the molecular level, we conducted a comprehensive genomic characterization using whole-genome sequencing (WGS). This high-resolution approach is essential for identifying potential genetic determinants associated with pathogenicity or transmissible antimicrobial resistance that may not be apparent through phenotypic testing alone (EFSA Scientific Committee, 2024).

**Fig. 1.**
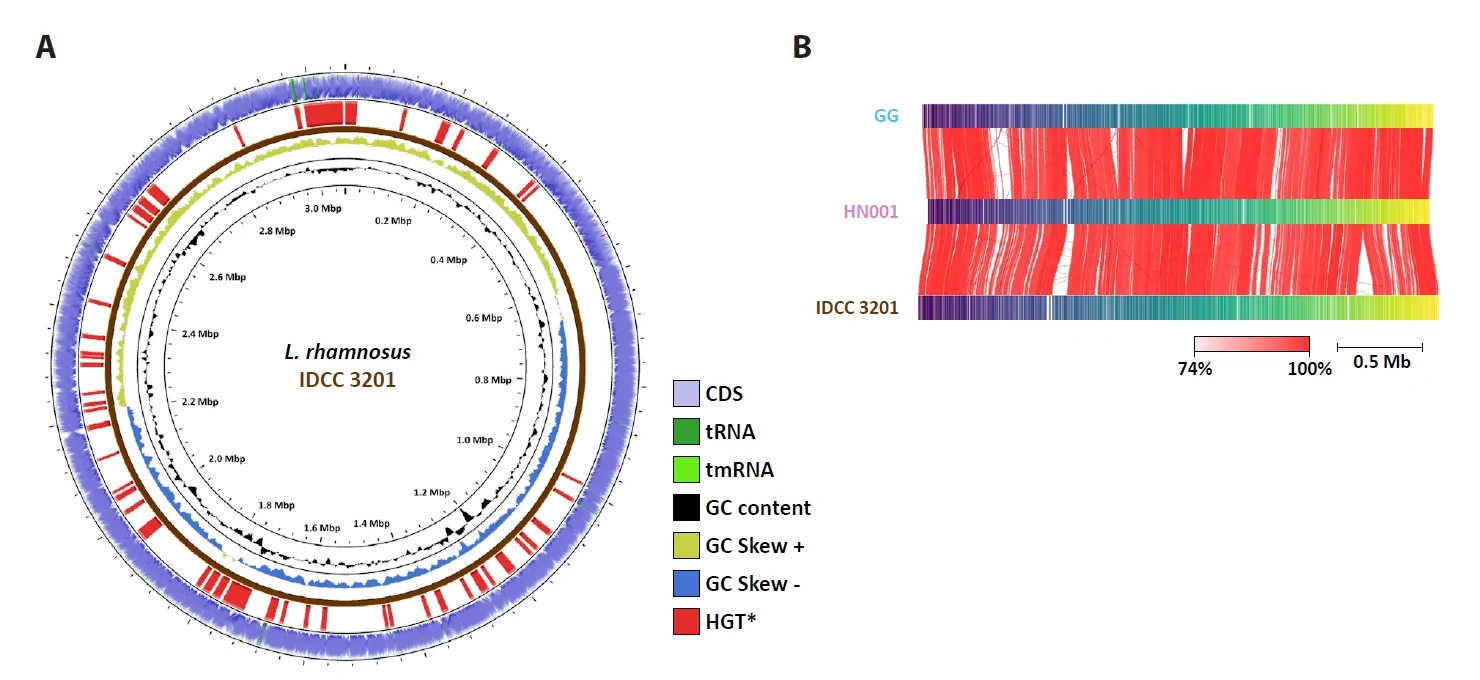
Genomic characterization and comparative genome analysis of *Lacticaseibacillus rhamnosus* IDCC 3201. (A) Circular genome map of the *L. rhamnosus* IDCC 3201 chromosome. The illustrated circular representation delineates the structural features of the ~3.1 Mb chromosome. The map displays genomic coordinates in Mb, protein-coding sequences (CDS) on the forward strand and the reverse strand, tRNA and tmRNA genes, GC content, GC skew and regions for horizontal gene transfer (HGT*). (B) Syntenic alignment of IDCC 3201 with the reference probiotic strains. A high-resolution linear synteny plot demonstrates the chromosomal congruence and divergence between *L. rhamnosus* IDCC 3201 and the well-known reference strains, *L. rhamnosus* GG and HN001. Red interconnecting lines indicate conserved orthologous gene blocks, showing substantial conservation of the core genome and identifying strain-specific genomic regions in IDCC 3201.

The WGS analysis revealed that the IDCC 3201 genome consists of a single circular chromosome of 3,064,263 bp with a G + C content of 46.69%, which aligns with the established genomic architecture of the *L. rhamnosus* species (Fig. 1). To confirm its taxonomic identity and safety by association, we performed a comparative genomic analysis against two well-characterized probiotic strains: *L. rhamnosus* HN001 and GG. IDCC 3201 exhibited Average Nucleotide Identity (ANI) values of 98.01% (vs. HN001) and 98.25% (vs. GG), confirming its close phylogenetic relationship with these established probiotic strains (Table 1).

A critical safety requirement for probiotics is the absence of acquired or transferable antibiotic resistance genes (Sanders et al., 2010). To address this, we screened the IDCC 3201 genome against the Comprehensive Antibiotic Resistance Database (CARD). No acquired resistance genes associated with clinically relevant antimicrobials were detected. To validate these genomic findings, we conducted phenotypic antibiotic susceptibility testing in accordance with established regulatory guidelines (CLSI, 2026). IDCC 3201 demonstrated susceptibility to a broad spectrum of antibiotics, including ampicillin, erythromycin, clindamycin, tetracycline, and gentamicin. Notably, the Minimum Inhibitory Concentration (MIC) values were comparable to or lower than those of the reference strain *L. rhamnosus* HN001, indicating a lack of atypical resistance (Table 2).

To further ensure that IDCC 3201 does not harbor pathogenic potential, we systematically analyzed its genome using the Virulence Factors Database (VFDB) and VirulenceFinder. No genes associated with known pathogenic bacteria, such as *Escherichia coli*, *Enterococcus* spp., *Listeria monocytogenes*, or *Staphylococcus aureus*, were identified. While genomic screening provides a predictive baseline, we performed phenotypic assays to exclude the presence of harmful metabolic activities: we first confirmed that IDCC 3201 lacks hemolytic activity (i.e., γ-hemolysis) (Table S1), a finding consistent with our genomic predictions.

These genomic and phenotypic evaluations demonstrate that *L. rhamnosus* IDCC 3201 does not harbor genetic or metabolic traits associated with virulence, acquired antimicrobial resistance, or the production of toxic metabolites. These results establish a comprehensive safety profile for its use in probiotic applications.

### Genotoxicity assessment

**Fig. 2.**
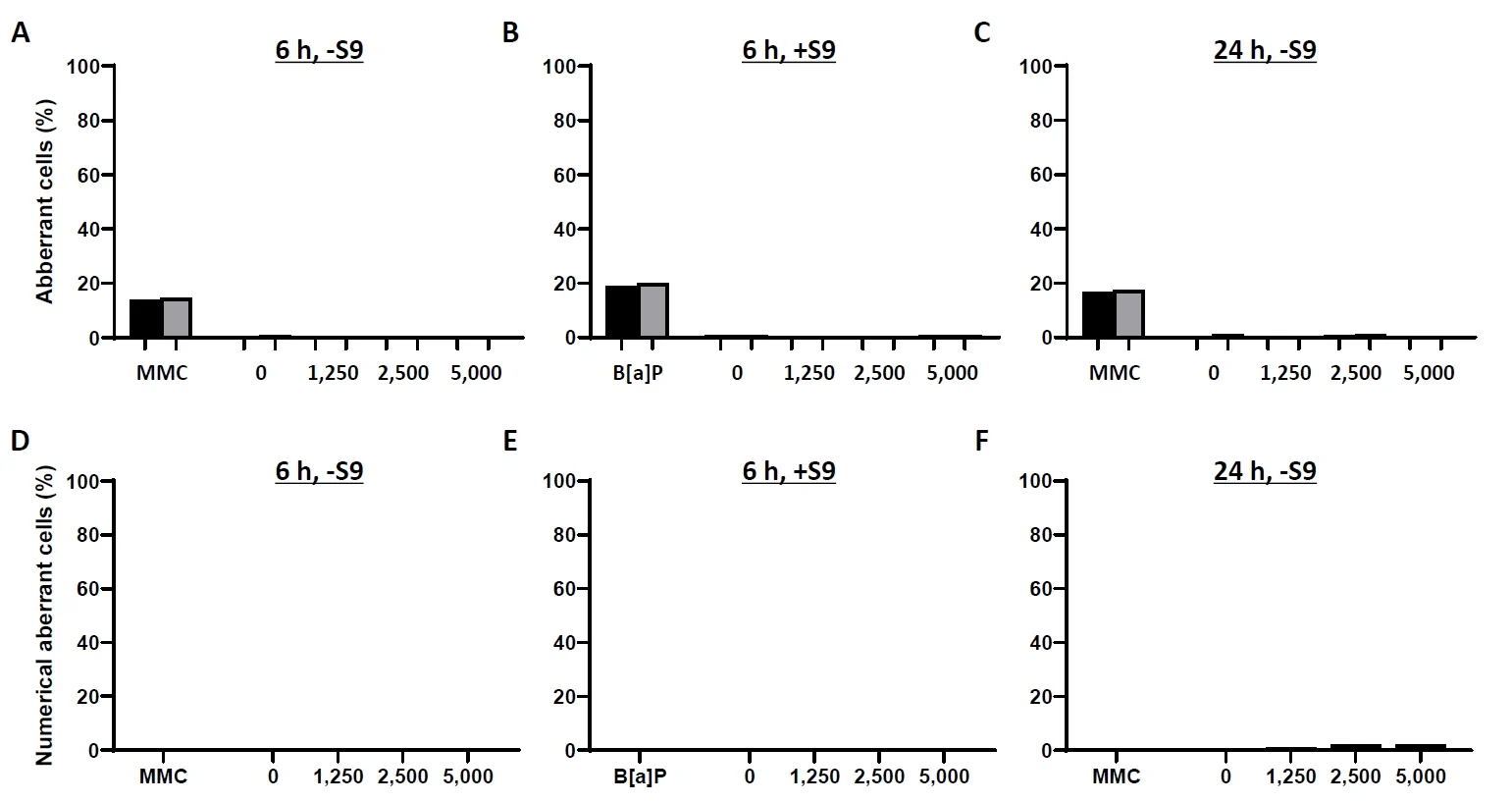
In vitro chromosomal aberration assay of RHT3201. The frequencies of cells with structural (A, B, and C) and numerical (D, E, and F) chromosomal aberrations were assessed in Chinese hamster lung (CHL/IU) cells following treatment with RHT3201 at the designated concentrations up to 5,000 μg/ml under short-term exposure (6 h) conditions in the absence (−S9) (A and D) or presence (+S9) (B and E) of metabolic activation, and under continuous exposure (24 h) conditions without metabolic activation (C and F). Percentages of the cells displaying structural aberrations excluding gaps (black bar) and including gaps (gray bar) are shown in panels A, B, and C, whereas frequencies of numerical aberrations (polyploidy) are shown in panels D, E, and F. Mitomycin C (MMC) (0.1 μg/ml for 6 h and 0.05 μg/ml for 24 h) and benzo[a]pyrene (B[a]P) (20 μg/ml for 6 h) were used as positive controls.

To ensure that RHT3201 does not pose a risk of hereditary damage or carcinogenic potential through DNA modification, we conducted a comprehensive genotoxicity assessment. Following international safety guidelines, we utilized a multi-tiered approach: an in vitro assay to detect cellular-level chromosomal damage and an in vivo study to evaluate systemic genetic stability in a mammalian model (OECD, 2016a, 2016b). We first performed an in vitro chromosomal aberration assay using Chinese Hamster Lung (CHL/IU) cells. Under all treatment conditions, RHT3201 demonstrated a high safety margin, with no evidence of cytotoxicity at concentrations up to 5,000 μg/ml. While the positive controls (mitomycin C and benzo[a]pyrene) induced the expected significant increases in chromosomal aberrations, RHT3201-treated cells showed no statistically significant differences compared to the vehicle control (Fig. 2). These results suggest that RHT3201 does not interfere with chromosomal integrity at the cellular level.

**Fig. 3.**
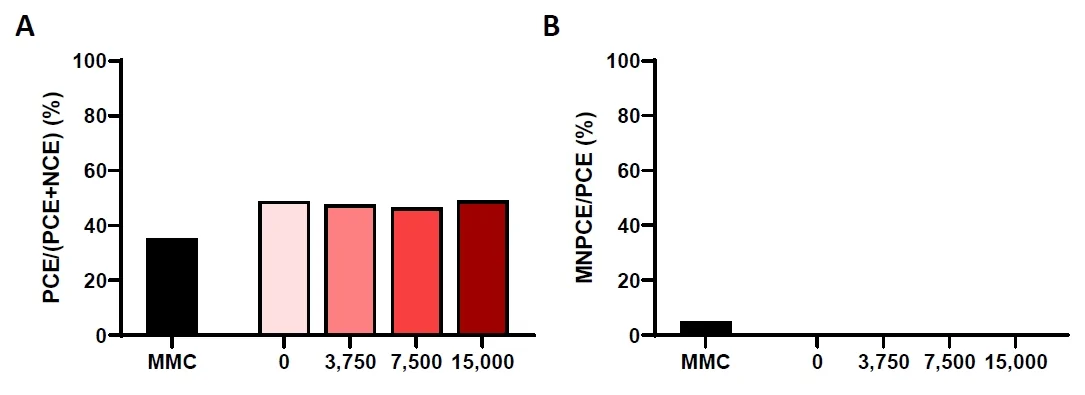
In vivo bone marrow micronucleus assay for RHT3201 in male mice. Bone marrow micronucleus formation was evaluated following oral administration of RHT3201 at doses up to 15,000 mg/kg/day. The ratio of polychromatic erythrocytes [PCE/(PCE+NCE)] (A) and the frequency of micronucleated polychromatic erythrocytes (MNPCEs) (B) were determined in bone marrow cells collected 24 h after the final administration. Mitomycin C (MMC) (2 mg/kg/day) was used as the positive control.

While in vitro results provide a sensitive baseline, evaluating genotoxicity in a whole-animal model is essential to account for mammalian metabolism, distribution, and the complex environment of bone marrow erythropoiesis. Consequently, we performed an in vivo micronucleus assay using ICR mice to detect clastogenicity (chromosomal breakage) or aneugenicity (damage to the mitotic apparatus). To determine if RHT3201 reached the target tissue and affected cell production, we monitored the proportion of polychromatic erythrocytes (PCE) relative to total erythrocytes (the PCE/(PCE+NCE) ratio). As shown in Fig. 3, this ratio did not decrease in a dose-dependent manner compared to the control group, indicating that RHT3201 does not inhibit bone marrow erythropoiesis or induce systemic cytotoxicity even at the doses up to 15,000 mg/kg/day administered twice at a 24-h interval. The definitive parameter for genetic damage in this model is the frequency of micronucleated polychromatic erythrocytes (MNPCEs). In contrast to the positive control (mitomycin C at 2 mg/kg/day), which produced significant genetic damage, RHT3201-treated mice showed no statistically significant or biologically relevant increase in MNPCEs. The lack of a dose-dependent trend in MNPCE frequency confirms that the compound does not induce chromosomal damage in vivo. The cumulative data from both in vitro chromosomal aberration and in vivo micronucleus assays demonstrate that RHT3201 is neither clastogenic nor aneugenic under the conditions tested. Based on these findings, we conclude that RHT3201 exhibits no genotoxic potential, supporting its safety for further therapeutic or commercial development.

### Oral toxicity assessment

**Fig. 4.**
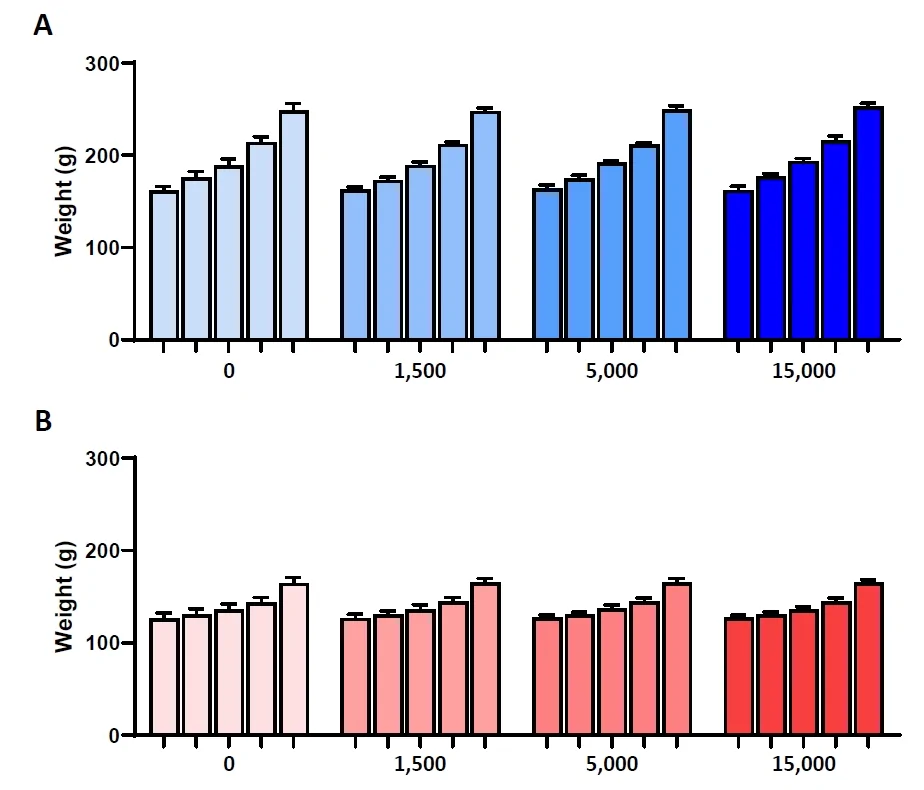
Body weights of male and female rats in the acute oral toxicity study of RHT3201. Body weights were monitored on days 0, 1, 3, 7, and 14 following a single oral administration of RHT3201 at the designated doses up to 15,000 mg/kg. Body weight changes in male (A) and female (B) Sprague-Dawley (SD) rats were evaluated over the 14-day post-administration observation period. The values are averaged from the three independent biological replicates and the error bars represent the standard deviations.

The oral toxicity of RHT3201 was evaluated through both acute and subchronic studies. To establish the baseline safety profile and determine the immediate physiological impact of high-dose exposure, we first conducted an acute oral toxicity study using Sprague-Dawley (SD) rats. This initial phase is critical for identifying potential target organs of toxicity and determining the upper safety limits for subsequent repeated-dose studies (OECD, 2008). To evaluate acute oral toxicity, RHT3201 was administered up to a maximum dose of 15,000 mg/kg delivered in two divided doses at a 2-h interval (Fig. 4). No significant differences in body weight alterations were observed between the negative control and the high-dose (15,000 mg/kg) groups for either male or female animals. Furthermore, no mortality or treatment-related clinical signs were recorded throughout the observation period. Although transient changes in stool consistency were noted in the maximum-dose groups, these symptoms were resolved spontaneously within 24 h. Collectively, the absence of significant body weight fluctuations or abnormalities during gross necropsy indicates that RHT3201 possesses a high margin of safety regarding acute oral exposure.

While the acute study confirmed that RHT3201 is well-tolerated at high single doses, evaluating the potential for cumulative toxicity and long-term systemic impact required a more rigorous, repeated-dose approach. Consequently, we initiated a 90-day subchronic toxicity study to monitor the physiological, biochemical, and histopathological effects of prolonged daily administration (OECD, 2018). RHT3201 was administered at doses up to 5,000 mg/kg/day. To assess general metabolic health, we monitored body weight and food consumption throughout the study (Fig. S1). These parameters remained largely comparable to the control group, with only negligible and transient decreases in food intake observed in the high-dose cohort, which did not impact overall growth. Furthermore, we conducted a suite of functional observations including visual, tactile, and auditory (click) responses to screen for potential neurobehavioral deficits. As shown in Table 3, no treatment-related adverse effects were observed in these functional assessments.

To gain deeper insight into the internal physiological state and detect subtle signs of organ dysfunction, we performed comprehensive hematological and biochemical analyses. These tests are essential for identifying subclinical changes in renal, hepatic, or hematopoietic function that might not manifest as overt clinical signs. Tables S2 and S3 indicated that all hematological properties and clinically significant biochemical indicators remained within normal physiological ranges, showing no significant deviation from the control group.

**Fig. 5.**
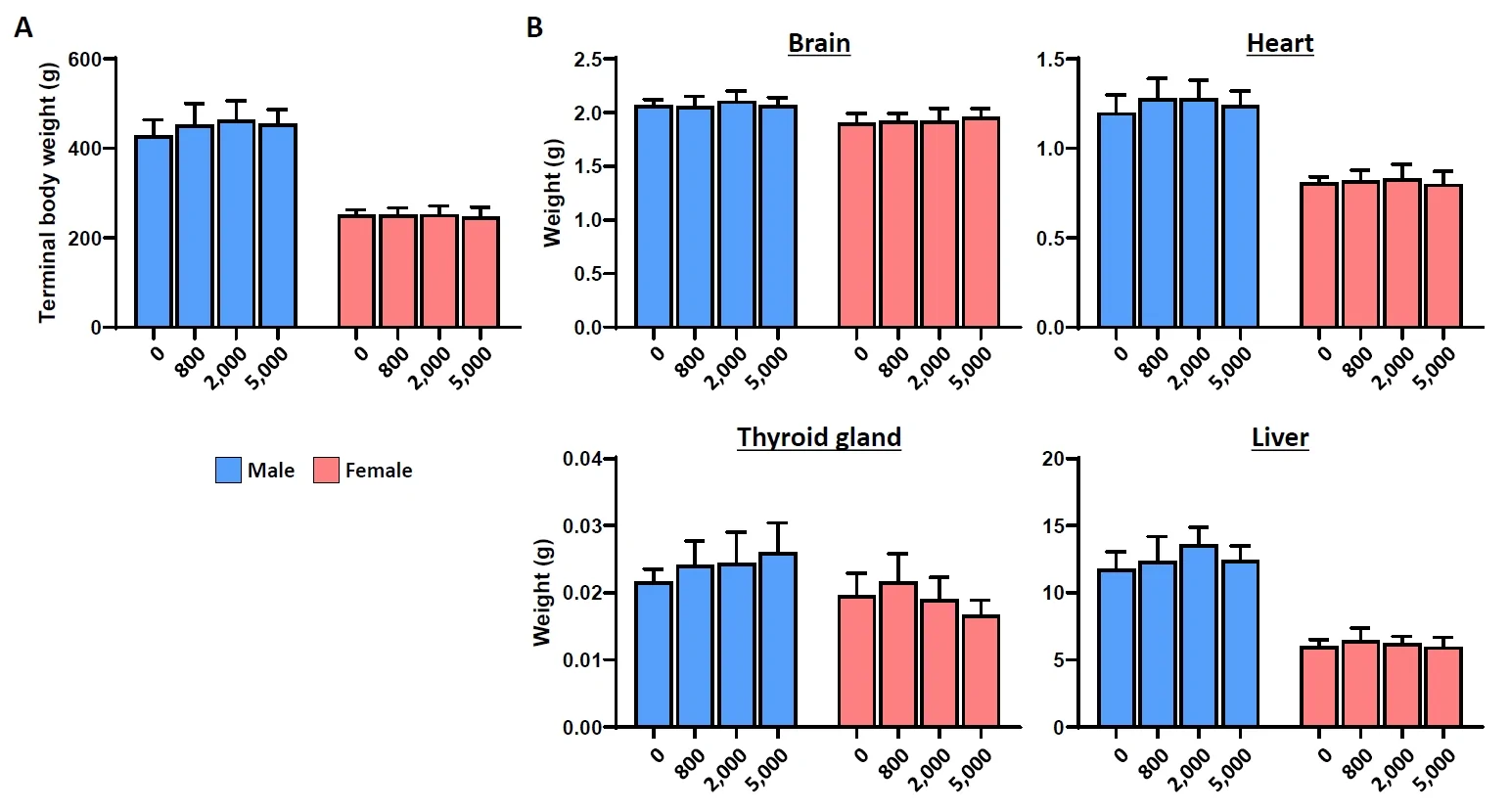
Terminal body weight and absolute organ weights of male and female rats in the 90-day subchronic oral toxicity study of RHT3201. Terminal body weight (A) and the weights of representative organs including brain (B), heart (C), thyroid gland (D), and liver (E) were evaluated following oral administration of RHT3201 at the designated doses up to 5,000 mg/kg/day for 90 days in male (blue) and female (red) SD rats (Table 4). The values are averaged from the three independent biological replicates and the error bars represent the standard deviations.

At the conclusion of the 90-day period, a terminal necropsy was performed to examine organ-specific effects. The absence of significant differences in organ weights (Fig. 5, Table 4) and the lack of macroscopic findings (Table S4) further supported the systemic safety of the compound. Finally, to ensure that no cellular-level damage was present, we conducted a thorough histopathological examination. This analysis revealed only minor, adaptive, and reversible changes that were deemed non-adverse (Table S5). Based on the cumulative evidence from both physiological monitoring and detailed pathological examinations, we concluded that repeated daily exposure to RHT3201 does not induce systemic toxicity. Consequently, the no-observed-adverse-effect-level (NOAEL) for RHT3201 was determined to be 5,000 mg/kg/day, the highest dose tested. These safety profiles support the continued development and potential therapeutic application of RHT3201.[Table t5-jm-2605002][Table t6-jm-2605002]

## Discussion

Postbiotics have emerged as a compelling alternative to conventional probiotics, primarily due to their superior physicochemical stability and the mitigation of safety risks inherently associated with the administration of viable microorganisms (Aguilar-Toalá et al., 2018; Salminen et al., 2021; Zhao et al., 2024). In the present study, we conducted a comprehensive toxicological evaluation of RHT3201, a postbiotic derived from *L. rhamnosus* IDCC 3201, incorporating genomic characterization, in vitro genotoxicity assays, and in vivo subchronic oral toxicity assessments. Our findings consistently demonstrate an absence of mutagenic, clastogenic, or systemic toxicity, thereby substantiating the safety profile of RHT3201 for inclusion as a functional ingredient in human applications.

In addition to safety profiles in this study, the immunological properties of *L. rhamnosus* IDCC 3201 and RHT3201 have been previously investigated (Jeong et al., 2020; Kim et al., 2017; Lee et al., 2016). Preclinical studies have demonstrated that RHT3201 retains immunomodulatory activity, including regulation of cytokine production and modulation of Th1/Th2 balance (Kim et al., 2017; Plaza-Díaz et al., 2017; Taverniti and Guglielmetti, 2011). Furthermore, clinical studies in human subjects have reported beneficial effects, such as reduced serum IgE levels and improvement of allergic symptoms, without significant safety concerns (Jeong et al., 2020). These findings support the notion that RHT3201 can be advantageous over the probiotics, based on the enhanced stability during processing and storage supports its application in functional foods and related products. The safety profile observed in this study is consistent with the previously reported clinical findings and its regulatory approval as an individually recognized functional ingredient in Korea. Nevertheless, further investigations, including long-term exposure studies, would be valuable to strengthen the safety assessment under chronic conditions. Importantly, RHT3201 has already been developed as a commercial product, and its safety can be further supported through continuous quality control and post-market surveillance. Such practices ensure consistent product quality and enable ongoing confirmation of its safety profile under real-world conditions (FEEDAP, 2012; Sanders et al., 2010).

Despite the robust safety profiles established in this study, several limitations necessitate further investigation. First, while our 90-day subchronic toxicity assessment provided essential safety benchmarks, it does not fully encapsulate the physiological implications of long-term, multi-year exposure. Subsequent longitudinal studies focusing on chronic toxicity would be invaluable to further validate the safety of RHT3201 under conditions of sustained consumption. Given that RHT3201 is already accessible as a commercial product, these efforts should be supplemented by rigorous post-market surveillance and continuous quality control to ensure product consistency and safety in diverse real-world populations (EFSA Scientific Committee, 2025; Sanders et al., 2010). Second, a significant challenge remains in the molecular characterization of the preparation. As postbiotics comprise a heterogeneous mixture of inactivated cellular structures and a complex array of metabolites, the specific bioactive molecular entities responsible for the observed functional effects have not yet been fully isolated or characterized (Aguilar-Toalá et al., 2018; Wegh et al., 2019). Although our study confirms the safety of the whole preparation, future research utilizing advanced metabolomics and proteomic profiling is required to deconstruct this complexity. Identifying these individual constituents will not only refine our understanding of the underlying mechanisms of action but also facilitate a more granular safety assessment at the level of specific bioactive components. Nevertheless, the integration of genomic, genotoxic, and systemic toxicity data presented here provides a definitive safety framework for RHT3201, supporting its continued development and application as a safe, stable, and efficacious functional agent.

## Figures and Tables

**Table 1.** Genomic information of IDCC 3201 and other two *Lacticaseibacillus rhamnosus* strains

Strains	IDCC 3201	HN001	GG
No. of contifgs	1	2	1
Plasmids	0	1	0
Genome size (bp^a^)	3,064,263	2,992,974	3,010,111
DNA G + C^b^ content (%)	46.69	46.76	46.69
No. of CDSs^c^	2,942	2,769	2,849
No. of rRNA genes	15	15	15
No. of tRNA genes	60	59	57
Mean of CDS lengths (bp)	836.9	903.1	851.2
Median of CDS lengths (bp)	717	786	738
Mean of intergenic lengths (bp)	137.2	154.9	131.4
Median of intergenic lengths (bp)	83	99	85
Homology with IDCC 3201 by OrthoANI^e^	NA^f^	98.01%	98.25%
Homology with IDCC 3201 by TNA^f^	NA	99.85%	99.82%

^a^bp = base pair; ^b^G + C = guanine + cytosine; ^c^CDSs = coding sequences; ^d^ANI = average nucleotide identity; ^e^TNA = tetra-nucleotide analysis; ^f^NA = not applicable.

**Table 2.** Susceptibility of the test strains to 9 antibiotics. Concentrations given as MIC in μg/ml

Strain	Em	Gm	Ap	Tc	Ca	Sm	Cm	Km	Vm
FEEDAP (2012)	1	16	4	8	4	32	1	64	n.r.^a^
SCAN (2003)	4	1	2^b^	16	16	16	-	32	4^b^
Danielson and Wind (2003)	1	128	4	4	16	> 256	2	> 256	-
Korhonen et al. (2010)	0.5	16	8	4	-	32	1	-	-
*L. rhamnosus* IDCC 3201	0.5	16	0.25	0.25	8	32	0.5	256	> 256
*L. rhamnosus* HN001	0.5	64	0.5	1	8	128	1	256	> 256
*L. rhamnosus* GG^c^	0.25	0.25	1	2	8	64	-	-	> 128

Values are Mean. Em, erythromycin; Gm, gentamicin; Ap, ampicillin; Tc, tetracycline; Ca, chloramphenicol; Sm, streptomycin; Cm, clindamycin; Km, kanamycin; Vm, vancomycin. ^a^n.r., not required; ^b^Note by SCAN - Certain species are inherently resistant; ^c^Ji et al. (2003).

**Table 3.** Chromosome aberration assay results for CHL/IU cells treated with RHT3201

Test item	Concentration (μg/ml)	RPD (%)	Percent of structural aberrant cells excluding gaps (%)^a^	Percent of structural aberrant cells including gaps (%)^a^	Percent of numerical aberrant cells (%)^a^
6 h treatment without metabolic activation (6 h, -S9)					
RHT3201	0	100	0.667	1.000	0.000
	1,250	97.1	0.333	0.333	0.333
	2,500	98.9	0.000	0.333	0.000
	5,000	74.2	0.333	0.667	0.667
MMC	0.1	49.7	14.0^*^	14.7	0.333
6 h treatment with metabolic activation (6 h, +S9)					
RHT3201	0	100	0.667	0.667	0.000
	1,250	91.8	0.333	0.333	0.667
	2,500	92.4	0.000	0.000	0.333
	5,000	91.1	0.667	0.667	0.000
B[a]P	20	48.8	19.0^*^	20.0	0.667
24 h treatment without metabolic activation (24 h, -S9)					
RHT3201	0	100	0.333	1.000	0.667
	1,250	94.7	0.000	0.000	1.000
	2,500	99.1	0.667	1.000	2.000
	5,000	90.6	0.000	0.000	2.000
MMC	0.05	72.6	16.7^*^	17.3	0.000

Values are Mean. MMC, mitomycin C; B[a]P, benzo[a]pyrene; RPD, relative population doubling. ^a^Mean percentage of duplicate culture; total 300 metaphase cells were examined (150 cells/culture). ^*^Significantly different from the vehicle control at *P* < 0.01.

**Table 4.** Micronucleus assay results for ICR mice administered with RHT3201

Test item	Dose (mg/kg/day)	Percent of PCE/(PCE + NCE) (%)	Percent of MNPCE/PCE (%)
RHT3201	0	48.9 ± 0.91	0.075 ± 0.047
	3,750	47.6 ± 3.69	0.070 ± 0.041
	7,500	46.5 ± 3.84	0.095 ± 0.057
	15,000	49.0 ± 3.77	0.055 ± 0.048
MMC	2	35.2^*^ ± 1.52	4.855^*^ ± 0.359

Values are Mean ± SD. MMC, mitomycin C; PCE, polychromatic erythrocyte; NCE, normochromatic erythrocyte; MNPCE, micronucleated polychromatic erythrocyte. *Significantly different from the vehicle control at *P* < 0.01.

**Table 5. t5-jm-2605002:** Functional observation battery results for SD rats administered with RHT3201 for 90 days

Dose (mg/kg/day)	Visual response	Touch response		Click response		Tail pinch response		Aerial righting reflex		Hindlimb landing foot splay (mm)		Forelimb grip strength (g)		Hindlimb grip strength (g)
Males														
0	3 ± 0	3 ± 0		3 ± 0		3 ± 0		0 ± 0		65.24 ± 18.33		1385 ± 112		800 ± 54
800	3 ± 0	3 ± 0		3 ± 0		3 ± 0		0 ± 0		68.98 ± 20.14		1414 ± 149		756 ± 113
2,000	3 ± 0	3 ± 0		3 ± 0		3 ± 0		0 ± 0		70.75 ± 18.17		1488 ± 174		762 ± 62
5,000	3 ± 0	3 ± 0		3 ± 0		3 ± 0		0 ± 0		66.74 ± 12.13		1459 ± 145		745 ± 86
**Dose (mg/kg/day)**	**Ambulatory counts (minutes interval)**													
	**0–10**		**10–20**		**20–30**		**30–40**		**40–50**		**50–60**		**Total**	
Males														
0	2020 ± 502		1156 ± 478		843 ± 669		526 ± 317		364 ± 316		437 ± 403		5346 ± 1770	
800	1777^*^ ± 376		1254 ± 436		897 ± 238		575 ± 328		424 ± 301		289 ± 312		5216 ± 1528	
2,000	2399^*^ ± 678		1422 ± 423		892 ± 417		758 ± 389		459 ± 564		327 ± 704		6256 ± 1936	
5,000	2308 ± 572		1296 ± 477		673 ± 315		307 ± 335		157 ± 194		259 ± 286		5000 ± 1357	
**Dose (mg/kg/day)**	**Vertical counts (minutes interval)**													
	**0–10**		**10–20**		**20–30**		**30–40**		**40–50**		**50–60**		**Total**	
Males														
0	115 ± 25		88 ± 30		67 ± 35		54 ± 32		49 ± 46		51 ± 42		424 ± 157	
800	126 ± 36		98 ± 31		78 ± 31		56 ± 25		44 ± 23		31 ± 28		432 ± 124	
2,000	108 ± 24		90 ± 22		59 ± 22		49 ± 23		34 ± 31		20 ± 31		359 ± 99	
5,000	107 ± 43		72 ± 36		48 ± 26		33 ± 29		15 ± 19		16 ± 18		291 ± 128	
**Dose (mg/kg/day)**	**Visual response**	**Touch response**		**Click response**		**Tail pinch response**		**Aerial righting reflex**		**Hindlimb landing foot splay (mm)**		**Forelimb grip strength (g)**		**Hindlimb grip strength (g)**
Females														
0	3 ± 0	3 ± 0		3 ± 0		3 ± 0		0 ± 0		67.61 ± 12.21		1261 ± 140		607 ± 58
800	3 ± 0	3 ± 0		3 ± 0		3 ± 0		0 ± 0		61.66 ± 16.64		1277 ± 125		564 ± 89
2,000	3 ± 0	3 ± 0		3 ± 0		3 ± 0		0 ± 0		62.42 ± 16.30		1303 ± 116		627 ± 53
5,000	3 ± 0	3 ± 0		3 ± 0		3 ± 0		0 ± 0		62.14 ± 19.73		1218 ± 148		626 ± 67
**Dose (mg/kg/day)**	**Ambulatory counts (minutes interval)**													
	**0–10**		**10–20**		**20–30**		**30–40**		**40–50**		**50–60**		**Total**	
Females														
0	2559 ± 377		1496 ± 420		1221 ± 438		816 ± 432		542 ± 410		514 ± 304		7147 ± 1658	
800	2370 ± 486		1498 ± 433		941 ± 239		568 ± 294		434 ± 358		530 ± 415		6342 ± 1174	
2,000	2320 ± 478		1696 ± 448		1232 ± 405		937 ± 477		788 ± 567		478 ± 475		7451 ± 2223	
5,000	2330 ± 623		1564 ± 440		950 ± 488		589 ± 422		641 ± 429		336 ± 306		6410 ± 1848	
**Dose (mg/kg/day)**	**Vertical counts (minutes interval)**													
	**0–10**		**10–20**		**20–30**		**30–40**		**40–50**		**50–60**		**Total**	
Females														
0	110 ± 21		89 ± 28		64 ± 25		47 ± 31		35 ± 22		32 ± 22		378 ± 124	
800	130 ± 19		93 ± 17		67 ± 22		51 ± 30		41 ± 28		32 ± 23		414 ± 106	
2,000	121 ± 23		97 ± 22		73 ± 27		43 ± 22		55 ± 36		37 ± 30		426 ± 128	
5,000	103 ± 41		70 ± 29		42 ± 25		28 ± 22		39 ± 36		23 ± 20		305 ± 150	

Values are Mean ± SD. Visual response 3: the animal approaches slowly and smells a stimulating bar; Touch response 3: the animal turns around slowly; Click response 3: twitching of body; Tail pinch response 3: squeaking, turning back; Aerial righting reflex 0: normal (landing on four limbs). *Significantly different from the vehicle control at *P* < 0.05.

**Table 6. t6-jm-2605002:** Organ weight results for SD rats administered with RHT3201 for 90 days

Dose (mg/kg/day)	Terminal body weight (g)	Brain		Heart		Pituitary gland		
		g	%	g	%	g	%	
Males								
0	429.3 ± 34.4	2.07 ± 0.05	0.49 ± 0.04	1.20 ± 0.10	0.28 ± 0.03	0.0127 ± 0.0013	0.0030 ± 0.0003	
800	452.3 ± 48.6	2.06 ± 0.09	0.46 ± 0.04	1.28 ± 0.11	0.29 ± 0.02	0.0136 ± 0.0007	0.0031 ± 0.0003	
2,000	463.0 ± 44.0	2.11 ± 0.09	0.46 ± 0.03	1.28 ± 0.10	0.28 ± 0.02	0.0131 ± 0.0016	0.0028 ± 0.0002	
5,000	455.3 ± 31.8	2.07 ± 0.07	0.45 ± 0.04	1.24 ± 0.08	0.27 ± 0.02	0.0132 ± 0.0013	0.0029 ± 0.0003	
**Dose (mg/kg/day)**	**Liver**		**Spleen**		**Lung**		**Thyroid gland^a^**	
	**g**	**%**	**g**	**%**	**g**	**%**	**g**	**%**
Males								
0	11.79 ± 1.27	2.75 ± 0.21	0.76 ± 0.12	0.18 ± 0.05	1.59 ± 0.12	0.37 ± 0.03	0.0216 ± 0.0019	0.0051 ± 0.0005
800	12.34 ± 1.85	2.73 ± 0.27	0.75 ± 0.12	0.17 ± 0.02	1.65 ± 0.14	0.36 ± 0.02	0.0241 ± 0.0036	0.0054 ± 0.0010
2,000	13.60^*^ ± 1.27	2.95 ± 0.22	0.82 ± 0.15	0.18 ± 0.02	1.67 ± 0.18	0.36 ± 0.02	0.0244 ± 0.0046	0.0053 ± 0.0009
5,000	12.46 ± 1.07	2.74 ± 0.20	0.75 ± 0.08	0.16 ± 0.01	1.65 ± 0.09	0.36 ± 0.02	0.0260 ± 0.0044	0.0057 ± 0.0007
**Dose (mg/kg/day)**	**Kidney**		**Thymus**		**Testis**		**Adrenal gland**	
	**g**	**%**	**g**	**%**	**g**	**%**	**g**	**%**
Males								
0	2.69 ± 0.25	0.63 ± 0.05	0.30 ± 0.06	0.07 ± 0.01	3.79 ± 0.26	0.88 ± 0.06	0.0462 ± 0.0033	0.0108 ± 0.0011
800	2.74 ± 0.24	0.61 ± 0.05	0.30 ± 0.06	0.07 ± 0.01	4.00 ± 0.15	0.89 ± 0.09	0.0533^*^ ± 0.0082	0.0118 ± 0.0017
2,000	2.87 ± 0.22	0.62 ± 0.05	0.31 ± 0.09	0.07 ± 0.02	3.90 ± 0.37	0.85 ± 0.08	0.0494 ± 0.0070	0.0107 ± 0.0012
5,000	2.80 ± 0.24	0.62 ± 0.05	0.32 ± 0.09	0.07 ± 0.02	3.99 ± 0.24	0.88 ± 0.05	0.0510 ± 0.0134	0.0111 ± 0.0025
**Dose (mg/kg/day)**	**Epididymis**		**Prostate^b^**					
	**g**	**%**	**g**	**%**				
Males								
0	1.37 ± 0.16	0.32 ± 0.04	3.18 ± 0.38	0.74 ± 0.09				
800	1.46 ± 0.08	0.32 ± 0.03	3.27 ± 0.50	0.72 ± 0.09				
2,000	1.44 ± 0.17	0.31 ± 0.03	3.23 ± 0.29	0.70 ± 0.07				
5,000	1.43 ± 0.13	0.31 ± 0.01	3.18 ± 0.53	0.70 ± 0.10				
**Dose (mg/kg/day)**	**Terminal body weight (g)**	**Brain**		**Heart**		**Pituitary gland**		
		**g**	**%**	**g**	**%**	**g**	**%**	
Females								
0	251.0 ± 11.7	1.90 ± 0.09	0.76 ± 0.06	0.81 ± 0.03	0.32 ± 0.02	0.0158 ± 0.0021	0.0063 ± 0.0010	
800	250.6 ± 16.7	1.92 ± 0.07	0.77 ± 0.05	0.82 ± 0.06	0.33 ± 0.02	0.0166 ± 0.0029	0.0067 ± 0.0013	
2,000	252.2 ± 19.0	1.92 ± 0.12	0.76 ± 0.05	0.83 ± 0.08	0.33 ± 0.02	0.0169 ± 0.0029	0.0067 ± 0.0010	
5,000	246.2 ± 22.4	1.96 ± 0.08	0.80 ± 0.06	0.80 ± 0.07	0.33 ± 0.01	0.0155 ± 0.0019	0.0063 ± 0.0007	
**Dose (mg/kg/day)**	**Liver**		**Spleen**		**Lung**		**Thyroid gland^a^**	
	**g**	**%**	**g**	**%**	**g**	**%**	**g**	**%**
Females								
0	6.02 ± 0.48	2.40 ± 0.15	0.55 ± 0.08	0.22 ± 0.03	1.19 ± 0.06	0.47 ± 0.03	0.0196 ± 0.0033	0.0078 ± 0.0015
800	6.48 ± 0.88	2.58 ± 0.28	0.56 ± 0.08	0.22 ± 0.02	1.21 ± 0.09	0.48 ± 0.02	0.0216 ± 0.0042	0.0086 ± 0.0014
2,000	6.24 ± 0.52	2.48 ± 0.18	0.60 ± 0.11	0.24 ± 0.04	1.24 ± 0.13	0.49 ± 0.03	0.0190 ± 0.0033	0.0076 ± 0.0014
5,000	5.98 ± 0.70	2.42 ± 0.15	0.54 ± 0.04	0.22 ± 0.02	1.20 ± 0.09	0.49 ± 0.02	0.0167 ± 0.0022	0.0068 ± 0.0008
**Dose (mg/kg/day)**	**Kidney**		**Thymus**		**Uterus**		**Adrenal gland**	
	**g**	**%**	**g**	**%**	**g**	**%**	**g**	**%**
Females								
0	1.61 ± 0.11	0.64 ± 0.03	0.23 ± 0.05	0.09 ± 0.02	0.62 ± 0.26	0.24 ± 0.11	0.0624 ± 0.0107	0.0249 ± 0.0044
800	1.65 ± 0.11	0.66 ± 0.05	0.22 ± 0.04	0.09 ± 0.01	0.65 ± 0.17	0.26 ± 0.07	0.0663 ± 0.0082	0.0266 ± 0.0039
2,000	1.68 ± 0.16	0.67 ± 0.06	0.23 ± 0.08	0.09 ± 0.02	0.67 ± 0.23	0.26 ± 0.08	0.0699 ± 0.0071	0.0279 ± 0.0036
5,000	1.65 ± 0.20	0.67 ± 0.06	0.23 ± 0.03	0.09 ± 0.01	0.73 ± 0.24	0.30 ± 0.10	0.0636 ± 0.0087	0.0258 ± 0.0020
**Dose (mg/kg/day)**	**Ovary**							
	**g**	**%**						
Females								
0	0.0818 ± 0.0105	0.0326 ± 0.0040						
800	0.0828 ± 0.0115	0.0330 ± 0.0039						
2,000	0.0915 ± 0.0188	0.0362 ± 0.0064						
5,000	0.0869 ± 0.0181	0.0351 ± 0.0057						

Values are Mean ± SD.

a Organ weight for thyroid gland with parathyroid gland as a whole.

b Organ weight for prostate with seminal vesicle and coagulation gland as a whole.

* Significantly different from the vehicle control at *P* < 0.05.

## Supplementary Information

Table S1. Hemolysis of IDCC 3201

Table S2. Hematology and coagulation results for SD rats administered with RHT3201 for 90 days^a^

Table S3. Clinical chemistry results for SD rats administered with RHT3201 for 90 days^a^

Table S4. Macroscopic findings for SD rats administered with RHT3201 for 90 days^a^

Table S5. Microscopic findings for SD rats administered with RHT3201 for 90 days^a^

Fig. S1. Temporal changes in body weight and food consumption of SD rats in the 90-day subchronic oral toxicity study. Body weights (A and B) and food consumption (C and D) for male (A and C) and female (B and D) SD rats were monitored over the 90-day dosing period. RTH3201 was orally administered to SD rats at the following doses (mg/kg/day): 0 (empty circle), 800 (grey diamond), 2,000 (grey square), and 5,000 (filled circle). Data are presented as mean, with the error bars representing standard deviations. No statistically significant differences were observed across the treatment groups (*P* > 0.05).
