# Supplementary material for: Genotoxicity, acute and subchronic oral toxicity assessments of postbiotics of Lacticaseibacillus rhamnosus IDCC 3201
Source: J Microbiol. 2026 Jun 12;64(6):e2605002. doi: 10.71150/jm.2605002 (PMC13577071; doi:10.71150/jm.2605002)
Supplement: Supplementary file 1 [file jm-2605002-Supplementary-Fig-S1.pdf]

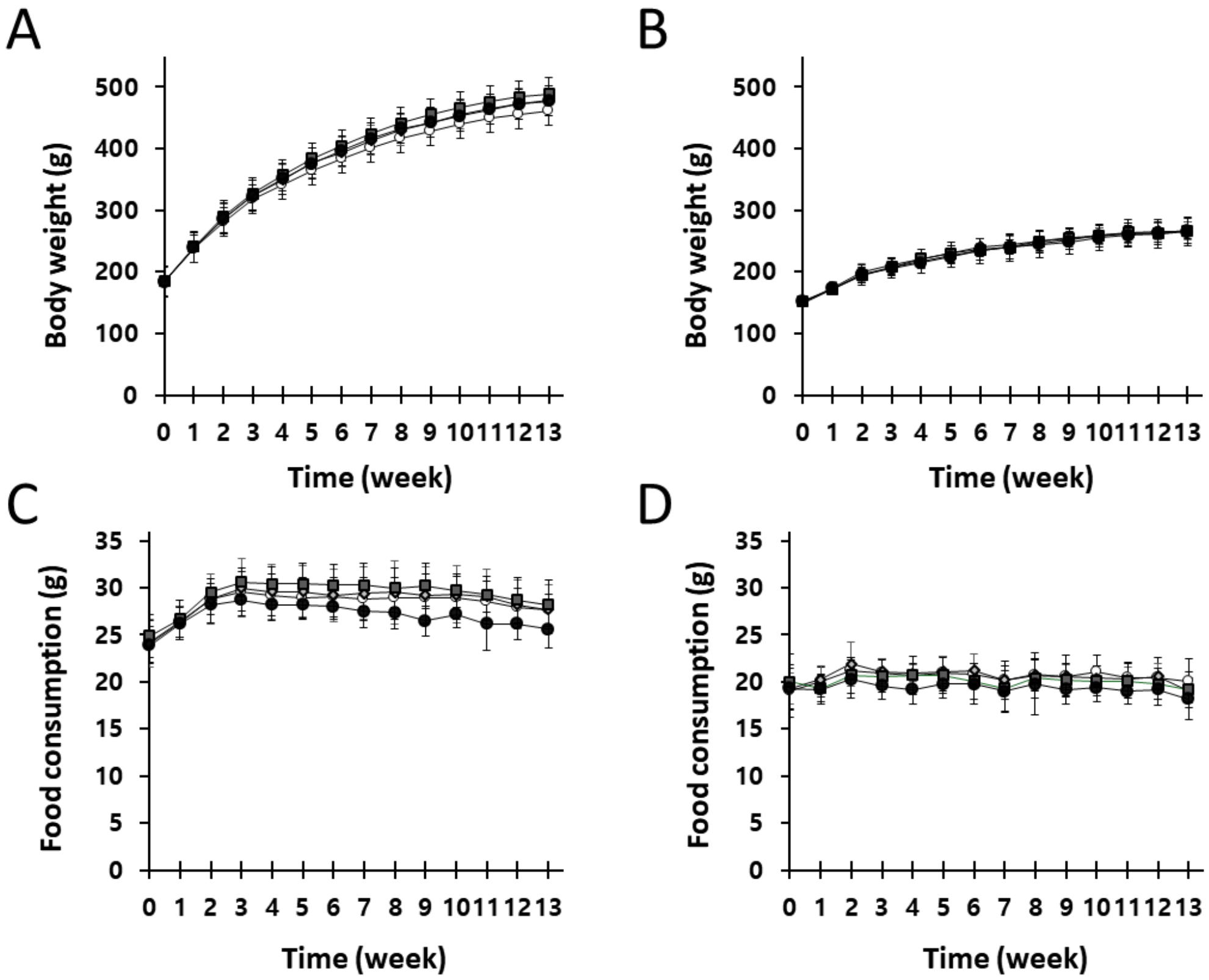

Fig. S1. Temporal changes in body weight and food consumption of SD rats in the 90-day subchronic oral toxicity study. Body weights (A and B) and food consumption (C and D) for male (A and C) and female (B and D) SD rats were monitored over the 90-day dosing period. RTH3201 was orally administered to SD rats at the following doses (mg/kg/day): 0 (empty circle), 800 (grey diamond), 2,000 (grey square), and 5,000 (filled circle). Data are presented as mean, with the error bars representing standard deviations. No statistically significant differences were observed across the treatment groups ($P > 0.05$).
