# Supplementary material for: Genotoxicity, acute and subchronic oral toxicity assessments of postbiotics of Lacticaseibacillus rhamnosus IDCC 3201
Source: J Microbiol. 2026 Jun 12;64(6):e2605002. doi: 10.71150/jm.2605002 (PMC13577071; doi:10.71150/jm.2605002)
Supplement: Supplementary file 2 [file jm-2605002-Supplementary-Table-S1.pdf]

Table S1. Hemolysis of IDCC 3201

Strain	Blood agar base No. 2	
	**Sheep**	**Horse**
Staphylococcus aureus ATCC 25923	β	β
Enterococcus faecalis ATCC 29212	β	β
Streptococcus pneumoniae ATCC 6305	α	α
Lacticaseibacillus rhamnosus IDCC 3201	γ	γ
L. rhamnosus HN001	γ	γ
L. rhamnosus GG	γ	γ
