# Supplementary material for: Genotoxicity, acute and subchronic oral toxicity assessments of postbiotics of Lacticaseibacillus rhamnosus IDCC 3201
Source: J Microbiol. 2026 Jun 12;64(6):e2605002. doi: 10.71150/jm.2605002 (PMC13577071; doi:10.71150/jm.2605002)
Supplement: Supplementary file 3 [file jm-2605002-Supplementary-Table-S2.pdf]

Table S2. Hematology and coagulation results for SD rats administered with RHT3201 for 90 days[a]

Dose	Male				Female			
(mg/kg/day)	0	800	2,000	5,000	0	800	2,000	5,000
RBC (10^6/μl)	8.82 ± 0.46	8.74 ± 0.30	8.72 ± 0.45	8.62 ± 0.22	7.96 ± 0.24	7.89 ± 0.28	8.07 ± 0.40	7.85 ± 0.29
HGB (g/dl)	15.8 ± 0.7	15.8 ± 0.6	15.7 ± 0.5	15.6 ± 0.4	15.1 ± 0.4	14.8 ± 0.5	15.2 ± 0.5	14.9 ± 0.6
HCT (%)	45.0 ± 1.8	44.8 ± 1.4	44.5 ± 1.5	44.3 ± 1.2	42.1 ± 0.8	41.6 ± 1.2	42.6 ± 1.6	41.8 ± 1.4
MCV (fL)	51.1 ± 1.8	51.2 ± 1.2	51.2 ± 1.7	51.4 ± 0.8	52.9 ± 1.6	52.8 ± 1.5	52.8 ± 1.7	53.3 ± 1.1
MCH (pg)	18.0 ± 0.7	18.1 ± 0.6	18.1 ± 0.7	18.1 ± 0.3	19.0 ± 0.7	18.8 ± 0.7	18.8 ± 0.8	19.0 ± 0.3
MCHC (g/dl)	35.2 ± 0.3	35.3 ± 0.6	35.3 ± 0.4	35.3 ± 0.3	35.9 ± 0.4	35.6 ± 0.3	35.7 ± 0.7	35.7 ± 0.3
PLT (10^3/μl)	1032 ± 87	1110 ± 90	1039 ± 117	948 ± 109	1030 ± 106	1079 ± 161	1111 ± 82	1062 ± 124
RET (10^6/μl)	0.1802 ± 0.0368	0.1891 ± 0.0257	0.1892 ± 0.0220	0.1849 ± 0.0158	0.1695 ± 0.0330	0.1658 ± 0.0224	0.1710 ± 0.0257	0.1869 ± 0.0324
WBC (10^3/μl)	6.30 ± 1.66	6.40 ± 1.26	6.62 ± 1.32	5.91 ± 1.70	2.68 ± 1.03	2.80 ± 0.80	2.74 ± 1.26	2.74 ± 0.66
NEU (10^3/μl)	1.49 ± 0.49	1.06 ± 0.40	1.00 ± 0.21	1.23 ± 1.06	0.37 ± 0.21	0.41 ± 0.11	0.36 ± 0.20	0.37 ± 0.13
LYM (10^3/μl)	4.17 ± 1.18	4.75 ± 1.24	4.94 ± 1.21	4.17 ± 1.01	2.06 ± 0.85	2.16 ± 0.66	2.12 ± 0.99	2.13 ± 0.58
MONO (10^3/μl)	0.47 ± 0.14	0.49 ± 0.12	0.55 ± 0.10	0.43 ± 0.15	0.18 ± 0.07	0.18 ± 0.08	0.20 ± 0.08	0.17 ± 0.06
EOS (10^3/μl)	0.15 ± 0.21	0.10 ± 0.04	0.12 ± 0.09	0.07 ± 0.02	0.06 ± 0.01	0.05 ± 0.04	0.06 ± 0.03	0.06 ± 0.04
BASO (10^3/μl)	0.01 ± 0.01	0.01 ± 0.01	0.01 ± 0.00	0.01 ± 0.01	0.00 ± 0.00	0.00 ± 0.00	0.00 ± 0.00	0.00 ± 0.00
PT (s)	8.6 ± 0.2	8.7 ± 0.3	8.6 ± 0.2	8.6 ± 0.2	8.0 ± 0.2	8.0 ± 0.2	7.7* ± 0.2	8.0 ± 0.2
APTT (s)	16.2 ± 1.1	16.4 ± 1.3	15.9 ± 0.8	16.2 ± 1.5	14.2 ± 1.2	14.7 ± 1.2	13.5 ± 1.3	13.9 ± 1.8
RET (%)	2.04 ± 0.34	2.17 ± 0.31	2.17 ± 0.21	2.15 ± 0.19	2.14 ± 0.46	2.11 ± 0.32	2.13 ± 0.35	2.39 ± 0.43
NEU (%)	23.8 ± 6.0	17.0 ± 8.1	15.5** ± 3.4	19.2 ± 11.7	14.2 ± 5.5	15.2 ± 3.9	13.5 ± 2.8	13.8 ± 4.2
LYM (%)	66.2 ± 7.6	73.7 ± 9.9	74.2 ± 4.5	72.2 ± 11.6	76.5 ± 6.0	76.6 ± 4.3	75.9 ± 6.9	77.4 ± 4.9

MONO (%)	7.6 ± 1.9	7.7 ± 1.7	8.4 ± 2.0	7.3 ± 1.5	6.9 ± 1.3	6.3 ± 0.9	7.9 ± 2.6	6.4 ± 2.0
EOS (%)	2.1 ± 2.5	1.5 ± 0.6	1.8 ± 1.0	1.2 ± 0.5	2.3 ± 0.7	1.8 ± 0.7	2.7 ± 2.2	2.3 ± 1.5
BASO (%)	0.2 ± 0.1	0.1 ± 0.1	0.2 ± 0.1	0.1 ± 0.1	0.1 ± 0.2	0.1 ± 0.1	0.0 ± 0.1	0.1 ± 0.1

[a]Values are Mean ± SD.

Abbreviations: RBC: red blood cell; HGB: hemoglobin; HCT: hematocrit; MCV: mean corpuscular volume; MCH: mean corpuscular hemoglobin; MCHC: mean corpuscular hemoglobin concentration; PLT: platelet; RET: reticulocyte; WBC: white blood cell; NEU: neutrophil; LYM: lymphocyte; MONO: monocyte; EOS: eosinophil; BASO: basophil; PT: prothrombin time; APTT: activated partial thromboplastin time.

Statistical significance: *, $P < 0.05$; **, $P < 0.01$.
