# Supplementary material for: Genotoxicity, acute and subchronic oral toxicity assessments of postbiotics of Lacticaseibacillus rhamnosus IDCC 3201
Source: J Microbiol. 2026 Jun 12;64(6):e2605002. doi: 10.71150/jm.2605002 (PMC13577071; doi:10.71150/jm.2605002)
Supplement: Supplementary file 4 [file jm-2605002-Supplementary-Table-S3.pdf]

Table S3. Clinical chemistry results for SD rats administered with RHT3201 for 90 days[a]

Dose	Male				Female			
(mg/kg/day)	0	800	2,000	5,000	0	800	2,000	5,000
AST (U/L)	62.5 ± 10.7	60.3 ± 6.7	64.5 ± 6.0	62.8 ± 10.4	61.6 ± 6.7	66.9 ± 10.6	69.1 ± 8.2	72.6 ± 18.5
ALT (U/L)	31.9 ± 3.5	30.0 ± 3.3	32.2 ± 6.5	31.3 ± 5.9	25.1 ± 4.5	30.6 ± 8.3	26.2 ± 4.9	34.3 ± 18.3
ALP (U/L)	239.8 ± 31.3	252.8 ± 46.1	258.2 ± 52.2	273.9 ± 69.1	168.8 ± 55.7	239.1 ± 179.3	183.7 ± 39.9	173.3 ± 48.2
GGT (U/L)	0.30 ± 0.27	0.12 ± 0.19	0.13 ± 0.13	0.11 ± 0.14	1.02 ± 0.43	1.19 ± 0.11	1.30 ± 0.48	1.60 ± 1.08
TBIL (mg/dl)	0.02 ± 0.01	0.02 ± 0.01	0.01 ± 0.02	0.02 ± 0.01	0.10 ± 0.03	0.09 ± 0.02	0.08 ± 0.02	0.08 ± 0.02
TCHO (mg/dl)	93 ± 15	86 ± 12	88 ± 17	78 ± 10	83 ± 10	87 ± 16	81 ± 10	74 ± 13
TG (mg/dl)	61 ± 35	54 ± 17	70 ± 24	50 ± 25	17 ± 6	16 ± 7	12 ± 3	16 ± 6
GLU (mg/dl)	182 ± 16	179 ± 18	183 ± 18	190 ± 22	131 ± 18	135 ± 20	122 ± 27	128 ± 14
BUN (mg/dl)	15.0 ± 1.4	15.1 ± 1.1	15.4 ± 3.0	15.5 ± 1.7	16.5 ± 2.1	17.9 ± 1.8	17.1 ± 1.4	16.7 ± 2.0
CREA (mg/dl)	0.43 ± 0.03	0.44 ± 0.06	0.43 ± 0.02	0.45 ± 0.05	0.48 ± 0.04	0.50 ± 0.05	0.51 ± 0.06	0.43 ± 0.09
TP (g/dl)	6.1 ± 0.2	6.1 ± 0.2	6.2 ± 0.2	6.0 ± 0.1	6.2 ± 0.3	6.1 ± 0.3	6.2 ± 0.2	6.0 ± 0.2
ALB (g/dl)	2.2 ± 0.1	2.2 ± 0.1	2.2 ± 0.1	2.2 ± 0.1	2.6 ± 0.2	2.5 ± 0.1	2.5 ± 0.2	2.5 ± 0.1
IP (mg/dl)	5.44 ± 0.36	5.32 ± 0.25	5.31 ± 0.31	5.54 ± 0.32	4.40 ± 0.52	4.72 ± 0.78	5.02 ± 0.68	4.98 ± 0.87
Ca (mg/dl)	9.9 ± 0.2	10.0 ± 0.2	10.0 ± 0.3	10.0 ± 0.2	11.6 ± 0.3	11.9 ± 0.6	11.8 ± 0.5	11.9 ± 0.4
HDL (mg/dl)	20.9 ± 1.8	20.0 ± 2.2	20.6 ± 2.8	19.4 ± 1.9	18.0 ± 1.6	19.5 ± 2.0	17.4 ± 1.5	17.0 ± 2.6
LDL (mg/dl)	9.7 ± 2.3	9.0 ± 1.4	7.4* ± 1.6	8.1 ± 2.0	4.6 ± 1.3	4.9 ± 1.9	5.1 ± 1.4	4.6 ± 1.1
TBA (μmol/L)	12.4 ± 9.0	9.1 ± 5.2	10.0 ± 5.6	9.4 ± 6.9	15.5 ± 13.5	9.7 ± 3.1	16.9 ± 10.9	16.7 ± 7.4
T3 (ng/ml)	0.567 ± 0.141	0.606 ± 0.148	0.608 ± 0.142	0.679 ± 0.174	0.505 ± 0.069	0.523 ± 0.122	0.557 ± 0.130	0.534 ± 0.080
T4 (ng/ml)	14.196 ± 2.380	16.781 ± 3.579	15.521 ± 5.241	23.622* ± 17.303	4.724 ± 1.650	5.093 ± 2.389	5.241 ± 1.477	5.416 ± 2.477

TSH (ng/ml)	6.944 ± 3.584	6.158 ± 2.062	8.018 ± 9.348	10.611 ± 8.035	7.806 ± 2.537	11.052 ± 5.444	9.358 ± 2.250	8.903 ± 3.553
Na (mmol/L)	144 ± 1	144 ± 1	144 ± 1	144 ± 1	143 ± 1	143 ± 1	144 ± 2	144 ± 1
K (mmol/L)	4.7 ± 0.2	4.6 ± 0.3	4.7 ± 0.4	4.7 ± 0.3	4.3 ± 0.2	4.3 ± 0.2	4.3 ± 0.5	4.3 ± 0.3
Cl (mmol/L)	104 ± 1	104 ± 1	103 ± 1	104 ± 1	105 ± 1	105 ± 1	105 ± 2	105 ± 2
A/G	0.58 ± 0.05	0.58 ± 0.03	0.57 ± 0.03	0.59 ± 0.03	0.70 ± 0.06	0.69 ± 0.03	0.68 ± 0.06	0.69 ± 0.04

[a]Values are Mean ± SD.

Abbreviations: AST: aspartate aminotransferase; ALT: alanine aminotransferase; ALP: alkaline phosphatase; GGT: gamma glutamyl transpeptidase; TBIL: total bilirubin; TCHO: total cholesterol; TG: triglyceride; GLU: glucose; BUN: blood urea nitrogen; CREA: creatinine; TP: total protein; ALB: albumin; IP: inorganic phosphorus; Ca: calcium; HDL: high density lipoprotein cholesterol; LDL: low density lipoprotein cholesterol; TBA: total bile acid; T3: triiodothyronine; T4: thyroxine; TSH: thyroid stimulating hormone; Na: sodium; K: potassium; Cl: chloride; A/G: albumin/globulin ratio.

Statistical significance: *, $P < 0.05$.
