# Supplementary material for: Genotoxicity, acute and subchronic oral toxicity assessments of postbiotics of Lacticaseibacillus rhamnosus IDCC 3201
Source: J Microbiol. 2026 Jun 12;64(6):e2605002. doi: 10.71150/jm.2605002 (PMC13577071; doi:10.71150/jm.2605002)
Supplement: Supplementary file 5 [file jm-2605002-Supplementary-Table-S4.pdf]

Table S4. Macroscopic findings for SD rats administered with RHT3201 for 90 days[a]

Organ/Tissue		Male		Female[b]
		Skin	Stomach	Stomach
Macroscopic finding		Nodule	Pitted	Pitted
Dose (mg/kg/day)	0	0	0	1
	800	0	0	0
	2,000	0	0	0
	5,000	1	1	0

[a]Data from the 10 animals examined; [b]No skin nodules observed in females.
