# Supplementary material for: Genotoxicity, acute and subchronic oral toxicity assessments of postbiotics of Lacticaseibacillus rhamnosus IDCC 3201
Source: J Microbiol. 2026 Jun 12;64(6):e2605002. doi: 10.71150/jm.2605002 (PMC13577071; doi:10.71150/jm.2605002)
Supplement: Supplementary file 6 [file jm-2605002-Supplementary-Table-S5.pdf]

Table S5. Microscopic findings for SD rats administered with RHT3201 for 90 days[a]

Organ		Male				Female	
		Liver[b]		Thyroid gland[c]		Liver[b]	Thyroid gland[c]
Microscopic finding		minimal	mild	minimal	mild	minimal[d]	minimal[d]
Dose (mg/kg/day)	0	5	0	3	0	0	0
	800	6	2	4	2	3	0
	2,000	4	5	6	1	4	0
	5,000	3	7	5	2	4	1

[a]Data from the 10 animals examined; [b]Centrilobular hepatocellular hypertrophy; [c]Diffuse follicular cell hypertrophy; [d]No mild changes observed in females.
